# Structural Modification in Anesthetic Drug Development for Prodrugs and Soft Drugs

**DOI:** 10.3389/fphar.2022.923353

**Published:** 2022-07-01

**Authors:** Chaoyi Deng, Jin Liu, Wensheng Zhang

**Affiliations:** ^1^ Department of Anesthesiology, West China Hospital, Sichuan University, Chengdu, China; ^2^ Laboratory of Anesthesia and Critical Care Medicine, National-Local Joint Engineering Research Center of Translational Medicine of Anesthesiology, West China Hospital, Sichuan University, Chengdu, China

**Keywords:** anesthetics, prodrugs, soft drugs, pharmacokinetic strategies, drug metabolism

## Abstract

Among the advancements in drug structural modifications, the increased focus on drug metabolic and pharmacokinetic properties in the anesthetic drug design process has led to significant developments. Drug metabolism also plays a key role in optimizing the pharmacokinetics, pharmacodynamics, and safety of drug molecules. Thus, in the field of anesthesiology, the applications of pharmacokinetic strategies are discussed in the context of sedatives, analgesics, and muscle relaxants. In this review, we summarize two approaches for structural optimization to develop anesthetic drugs, by designing prodrugs and soft drugs. Drugs that both failed and succeeded during the developmental stage are highlighted to illustrate how drug metabolism and pharmacokinetic optimization strategies may help improve their physical and chemical properties.

## Introduction

Drug discovery and development is an expensive process with a high failure rate commonly spanning an average of 12 years (DiMasi et al., 2010; Van Norman, 2016). Prior to the approval of compounds for human use, drug candidates are subjected to a series of *in vitro* and *in vivo* experiments examining possible efficacy and safety profiles. However, the overall failure rate in drug approval for decades has been reported to be 80–90% (Yamaguchi et al., 2021), with poor efficacy and/or unacceptable toxicity being the major causes of drug attrition at any developmental stage (Bass et al., 2009). Moreover, unforeseen toxicity accounts for 20–30% of clinical failure and remains one of the leading causes of drug recall and restriction (Berg, 2019).

During the early stages of drug discovery, screening of drug candidates is commonly undertaken to identify promising lead compounds (Roberts, 2018). After this, structural modification is carried out to improve the potency and specificity while often overlooking the pharmacokinetic (PK) parameters and toxicity at this stage (Drews, 2000). Although potency is a crucial indicator of a potential drug candidate, the PK properties are invariably affect the effectiveness of the drug. To mitigate this occurrence, understanding the interplay between PK and pharmacodynamics (PD) in therapeutic use is critical (Gabrielsson et al., 2009).

Over the past few years, the application of metabolism and PK optimization strategies in the drug design process has been gradually recognized to minimize potential safety liabilities (Buchwald, 2020; Cerny et al., 2020). The PK profile of a compound involves absorption, distribution, metabolism, and excretion (ADME), among which drug metabolism plays perhaps the most crucial role in drug development. Drug metabolism influences the pharmacological and toxicological effects and plays a key role in optimizing the PD, PK, and safety of drug molecules (Zhang and Tang, 2018). The basic principle of drug metabolism is a biotransformation process that enables efficient excretion of compounds from the body. In general, metabolic processes occur in the liver because of its high levels of metabolic enzymes (Patel et al., 2016). Although organisms have developed mechanisms for degrading and excreting foreign substances, metabolic pathways may result in reactive or toxic metabolic intermediate formation, especially through oxidative metabolism (Gillette, 1979; Attia, 2010).

**FIGURE 1:**
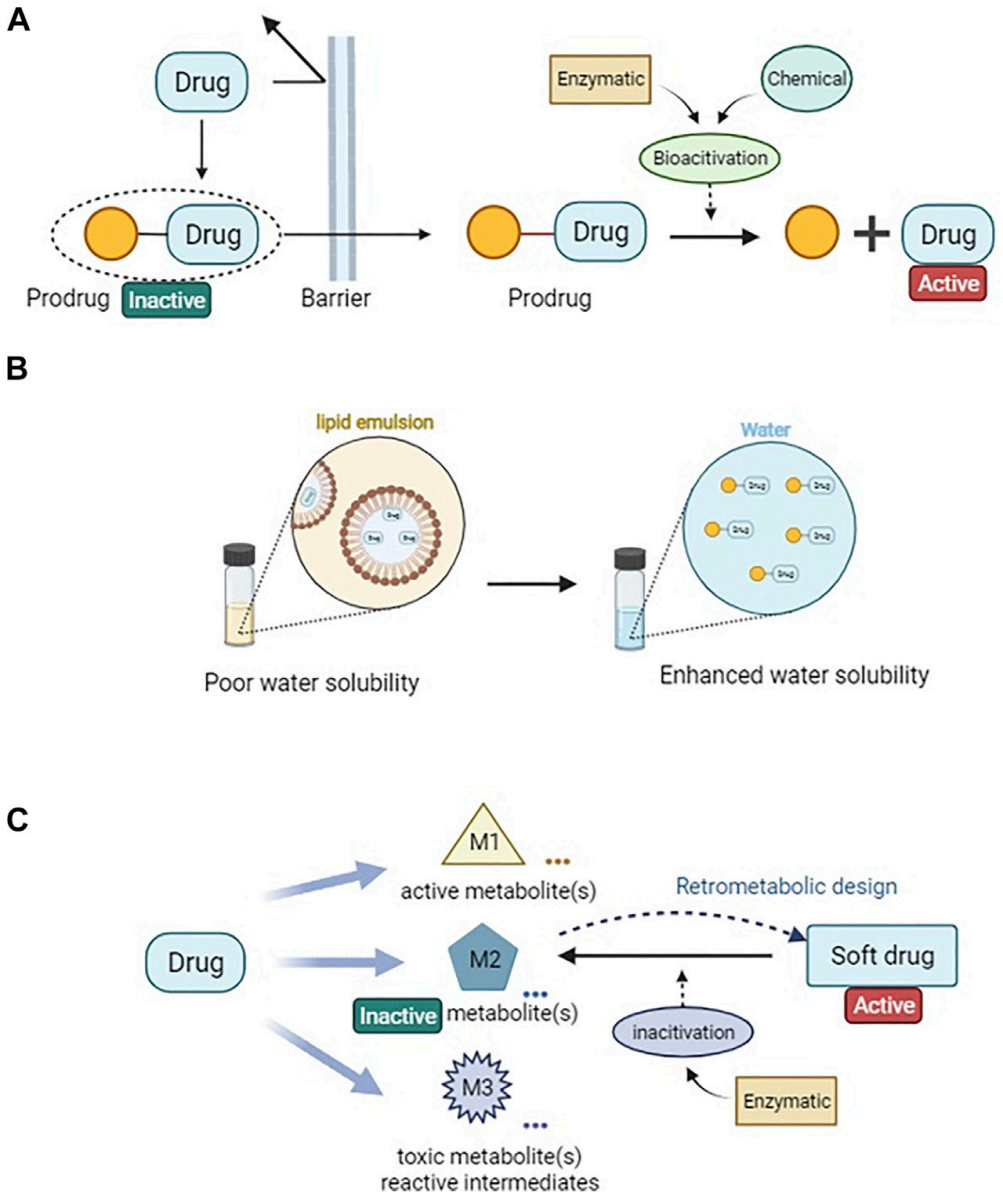
Drug metabolic and pharmacokinetic optimization strategies *via* prodrug and soft drug approach. **(A)** A simplified illustration of prodrug design for enhanced permeability. **(B)** Prodrug design for improving drug solubility. **(C)** A general sheme of soft drug design loop.

Studies on drug metabolism therefore play a key role in optimizing PK/PD properties and in reducing toxicity potential associated with bioactivation. It is desirable to design a “safer” drug focused on improving the activity/toxicity ratio, such as the therapeutic index, instead of improving activity alone (Bhardwaj et al., 2014). One approach is to design metabolically stable drugs, such as pharmacologically active compounds (e.g., hard drugs) with no or very limited metabolism. Such drugs are excreted by the body after they have exerted their therapeutic effects, thereby evading the problems associated with active intermediates or metabolites. Examples of successfully designed hard drugs are bisphosphonates and certain angiotensin-converting enzyme inhibitors (Kelly and O'Malley, 1990; Lin, 1996). Another approach is to integrate the structure–activity and structure–metabolism relationship of a drug to achieve controllable metabolism while improving its biological activity and therapeutic index (Bhardwaj et al., 2014). This can be achieved by the designing of prodrugs or soft drugs (Figure 1). In this review, we provide an overview of prodrug and soft drug design for improving PK/PD and safety profiles of anesthetic drugs.

## Prodrugs

Adrien Albert first introduced the term “prodrug” in 1958 (Albert, 1958). Prodrugs are pharmacologically inactive, bioreversible derivatives of active drug molecules. Prodrug designs commonly require the presence of functional groups, such as esters, amides, phosphates, carbonates, or carbamates, which are cleaved either enzymatically or chemically in the body (Rautio et al., 2008; Rautio et al., 2018).

The conversion of prodrugs to active drugs is distinct from the conversion of drugs to active metabolites. In the case of the former, the conversion of the pharmacologically inactive prodrugs to active drugs is designed with intended purposes. However, the conversion of drugs to active metabolites is an enzymatic process, and the sites of metabolism are unpredictable. While drugs and active metabolites are both pharmacologically active thus making it is an option to develop the active metabolites as new drugs, active metabolites can influence PK/PD relationships and pose considerable uncertainty in clinical trial (Anderson et al., 2009; Gabrielsson and Green, 2009).

**FIGURE 2:**
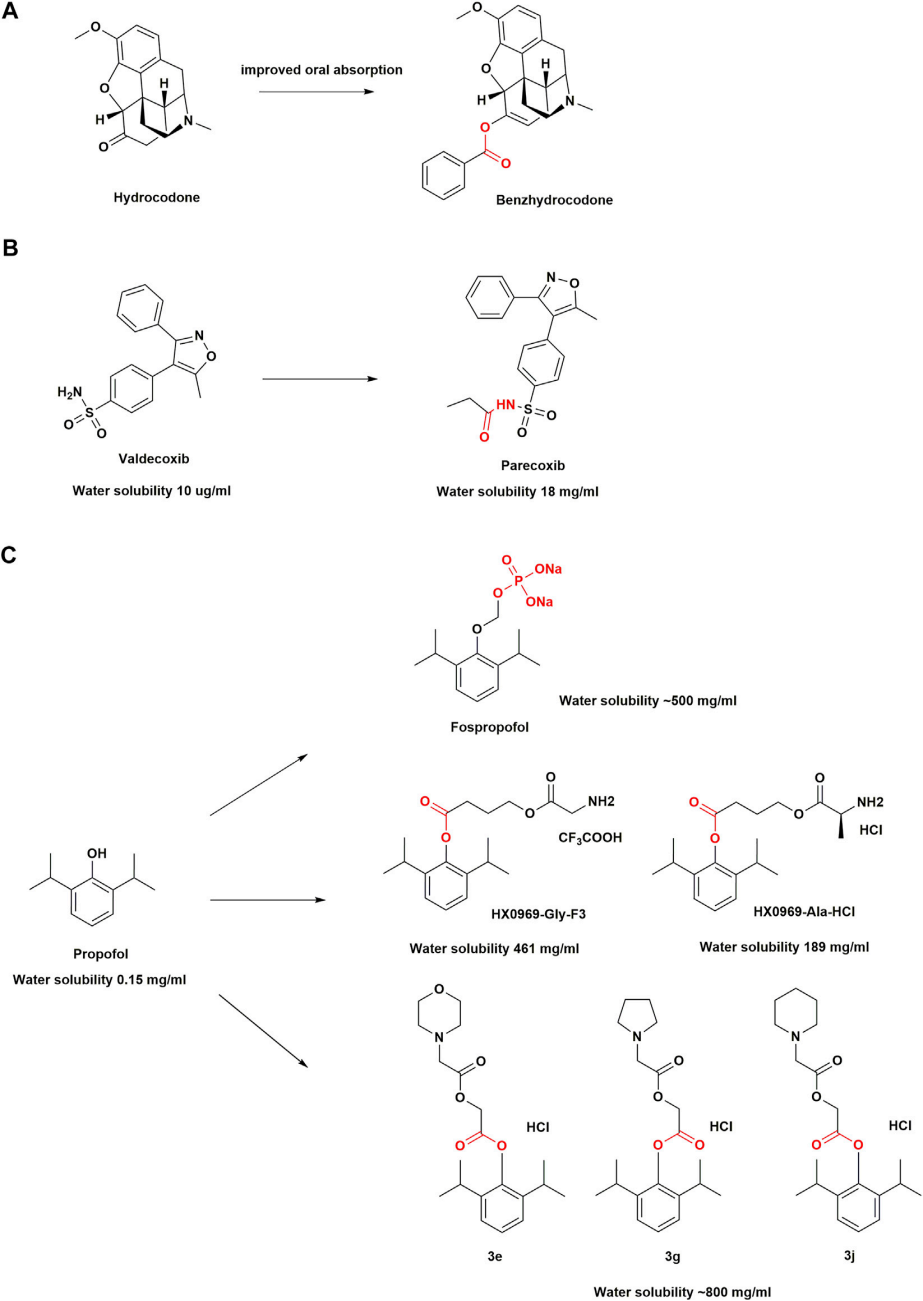
Structural optimization in anesthetic drugs with prodrug design. **(A)** Hydrocodone and its ester prodrug; **(B)** Valdecoxib and its amide prodrug; **(C)** Propofol prodrugs.

According to the type of carrier attached, prodrugs are conventionally classified into two major types: carrier-linked prodrugs and bio-precursors. Carrier-linked prodrugs have a non-toxic carrier or pro-moiety that is covalently linked and removed enzymatically to release the active drug moiety. In contrast, the bio-precursors do not incorporate a carrier group and only yield the active compounds upon biotransformation (Jornada et al., 2015). The prodrug strategy is often implemented to modify or eliminate undesirable physicochemical properties, such as poor solubility, limited bioavailability, chemical instability, low permeability, and lack of site-specificity, of pharmacologically active drug molecules. In other words, it enables optimization of absorption, distribution, metabolism, excretion, and toxicity (ADMET) properties of pharmacologically active moieties and overcomes formulation, delivery, and toxicity hurdles and achieve optimal drug therapy and outcomes (Rautio et al., 2018; Najjar et al., 2020). From 2008 to 2017, the United States Food and Drug Administration (FDA) approved at least 30 prodrugs, accounting for nearly 10% of all novel small-molecule compounds approved (Najjar and Karaman, 2019). Some successful strategies for prodrug design are described below as examples of prodrugs used in anesthesia (Figure 2).

## Ester Prodrugs

Ester prodrugs are most commonly used and it is estimated that around half of the marketed prodrugs are activated by enzyme-mediated hydrolysis (Ettmayer et al., 2004). The prodrug approach can mask polar or charged moieties as esters to improve lipophilicity, promote membrane permeation and enhance oral absorption (Beaumont et al., 2003). Moreover, The physicochemical properties are particularly suitable for CNS analgesia drugs (Kang et al., 2021).

Benzhydrocodone, a synthetic opioid, is a prodrug of hydrocodone (Mustafa et al., 2018). The physicochemical effect after adding benzoic acid functional groups to hydrocodone results in improved oral absorption and a reduction in parenteral bioavailability of the active metabolite (Cassidy et al., 2017; Guenther et al., 2018). Benzhydrocodone is inactive and exerts its pharmacologic effects mainly through the generation of hydrocodone, which has a high affinity for *µ*-opioid receptors (MORs). Upon binding, hydrocodone produces profound analgesia with no ceiling (Vallejo et al., 2011). It almost completely converted into hydrocodone within 5 min of oral administration, by esterase metabolism in the gastrointestinal tract (Silver et al., 2016). *In vitro* data have indicated that the conversion of benzhydrocodone to hydrocodone in whole blood is a slow process that takes approximately 240 min, which may deter parenteral abuse (Silver et al., 2016). A single-center, randomized, double-blind, crossover study among 51 healthy adults reported that intranasal administration of benzhydrocodone resulted in a significantly lower hydrocodone exposure and associated decrease in Drug Liking score compared with that of hydrocodone bitartrate (Mickle et al., 2018). In the case of prodrugs where transformation is required, a lower peak plasma concentration (C_max_) and a delayed time to peak the C_max_ compared with those for the parent drug may be observed (Mickle et al., 2018). In other words, a prodrug could potentially allow for better management of opioids toxicity. Benzhydrocodone in combination with acetaminophen (APAP) under the trade name Apadaz™ received FDA approval in February 2018 for the short-term management of severe acute pain (Mustafa et al., 2018). However, given that benzhydrocodone/APAP is still susceptible to oral abuse, it has not been confirmed as an abuse-deterrent opioid formulation.

## Amide Prodrugs

Amide prodrug derivatives are very common among nonsteroidal anti-inflammatory drugs (NSAIDs). Studies have shown that replacing the carboxylic group of NSAIDs with an amide functional group increases cyclooxygenase-2 (COX-2) selectivity and further helps to reduce the gastrointestinal toxicity of the parent drug (Kalgutkar et al., 2000).

Few NSAIDs were previously available for the parenteral treatment of acute and chronic pain, and their use was often accompanied by serious adverse effects, such as peptic ulcers, gastrointestinal bleeding, liver and kidney dysfunction, and platelet suppression (Pirani et al., 1987; Dorais et al., 1992; Strom et al., 1996). Therefore, an amide prodrug with high water solubility and anti-inflammatory activity to develop COX-2 inhibitors was designed for parenteral delivery. **Parecoxib** is the first parenteral and highly selective COX-2 inhibitor (Jain, 2000; Talley et al., 2000). As an amide prodrug of a sulfonamide-based COX-2 inhibitor valdecoxib, parecoxib are inactive. An N-acylation of the prodrug moiety of valdecoxib increases the water solubility of parecoxib, which makes the sulfonamide NH group more readily ionizable. The parecoxib amide hydrolysis of the sulfonyl propionamide substituent is mainly mediated by hepatic microsomal carboxylesterases (Talley et al., 2000), with a half-life of approximately 22 min. The onset of analgesic effects occurs within 10–23 min and attains a maximum relief within 2 h (Barton et al., 2002). Early clinical studies have shown that it brings pain relief in post-surgical patients *via* its rapid conversion to valdecoxib *in vivo* (Desjardins et al., 2001). A pooled analysis of 28 randomized, placebo-controlled clinical trials with 9287 patients has shown that skin rash and cardiac complications occur infrequently with parecoxib administration, which highlight its safety in patients (Schug et al., 2017). Currently, it is approved in over 80 countries for perioperative pain control and may help reduce opioid use (Diaz-Borjon et al., 2017).

## Phosphate Prodrugs

Prodrugs with ionizable functional groups (e.g., phosphate, phosphonate, and phosphinate) are designed to improve drug solubility, among which phosphate ester-based prodrugs are relatively stable and are a good substrate for alkaline phosphatases *in vivo* (Wiemer and Wiemer, 2015).

Propofol (2,6-diisopropylphenol) is an intravenous general anesthetic drug commonly used in clinical practice and is pharmacologically characterized by its rapid induction of anesthesia and recovery from it after discontinuation (Sahinovic et al., 2018). It is often formulated as an oil-in-water emulsion with high lipophilicity (150 μg/ml) (Rautio et al., 2008). The adverse effects associated with propofol emulsions, such as injection pain, bacterial contamination, and propofol infusion syndrome (Bennett et al., 1995; Kam and Cardone, 2007; Desousa, 2016), have led to the design of the drugs with improved water solubility, a hot spot in drug development. Therefore, phosphate prodrugs of propofol have been designed with a phosphate group attached to the hydroxyl group of propofol, such as fospropofol disodium, through an OCH_2_ spacer (Garnock-Jones and Scott, 2010). The substitution of the hydroxyl group by the charged phosphate group increases electronegativity, which greatly improves the water solubility of fospropofol (500 mg/ml) (Rautio et al., 2008). Once the phosphate group is hydrolyzed by alkaline phosphatase in the liver, liberating the active metabolite propofol, the resulting formaldehyde and phosphate degrade naturally (Schywalsky et al., 2003). Based on the molecular weight, 1 mg of fospropofol (332.24 g/mol) releases 0.54 mg of propofol (178.27 g/mol). Moreover, given that the “biophase” characteristics of fospropofol are different from that of propofol (Yavas et al., 2008), it results in a slower onset of the drug effect in the former group. Fospropofol has a sedation effect onset of 4–13 min and a prolonged effect duration. For prodrugs, due to the existence of enzymatic and/or chemical transformation processes, the therapeutic effects of the parent drug are usually delayed. Since the enzymatic conversion to propofol is time-dependent, fospropofol may theoretically provide a better safety profile, especially in cardiac and respiratory functions (Abdelmalak et al., 2012). For example, recent studies have shown a decreased incidence of hypotension and respiratory depression with fospropofol because of its slower onset of action (Luo et al., 2022). Moreover, contrary to what was observed in the administration of propofol, the oral and intraduodenal administration of fospropofol produced a propofol bioavailability of 30% or more in human volunteers (Wozniak et al., 2015). Therefore, the indications for propofol may be extended by nonintravenous administration of fospropofol.

Although fospropofol eliminates drawbacks associated with propofol emulsion in water-soluble formulations, common adverse events observed in patients are paresthesia (incidence 49–74%) and pruritus (incidence 16–28%), often in the perianal region (Cohen et al., 2010; Gan et al., 2010). The metabolic accumulation of phosphate components causes these adverse effects, which are transient and self-limited (Bengalorkar et al., 2011). Researchers have therefore also introduced amino acid groups into the design of propofol prodrugs. It was found that two modified amino acid prodrugs (HX0969-Ala-HCl and HX0969-Gly-F3) released propofol more rapidly than the phosphate prodrugs previously designed (fospropofol disodium and HX0969W), which was confirmed through *in vitro* plasma experiments in rats (Lang et al., 2014). *In vivo* experiments showed that the intravenous administration of amino acid prodrugs had a faster onset of action and required a lower dose than the phosphate prodrug, and prevented the generation of formaldehyde and phosphate, thereby eliminating the adverse effects associated with formaldehyde and phosphate buildup (Lang et al., 2014). This new design approach may improve the conversion efficiency of a prodrug.

Next, researchers found that the insertion of glycolic acid as a linker between propofol and cyclic amino acids could further accelerate the release of propofol into the plasma. Prodrugs (3e, 3g, 3j) have been shown to be better than fospropofol in terms of onset time, anesthesia duration time, and safety in mice (Liu et al., 2020). The molar mass, onset, and duration of action of these prodrugs were found to be comparable to those of propofol, while preserving the clinical benefits of propofol. In addition, propofol + glycolic acid + cyclic amino acids may yield a key structural feature that could contribute to the development of a safe, water-soluble, rapid-release propofol prodrug with high molecular utilization of propofol (Liu et al., 2020). However, because propofol is still to date the active ingredient in these prodrug designs, the adverse effects such as hypotension and respiratory depression remain unresolved.

## Soft Drugs

The term soft drug was introduced by Bodor during the late 1970s (Bodor et al., 1980; Bodor and Kaminski, 1980). Soft drugs are pharmacologically active and undergo predictable and controllable metabolic inactivation after exhibiting their therapeutic effect (Bodor and Buchwald, 2000). In general, soft drugs are designed to control metabolism and prevent the generation of potentially reactive or toxic intermediates. If possible, the inactivation process should occur in a single, low-energy, high-volume metabolic step in which the inactive substances produced are immediately eliminated (Buchwald and Bodor, 2014).

**FIGURE 3:**
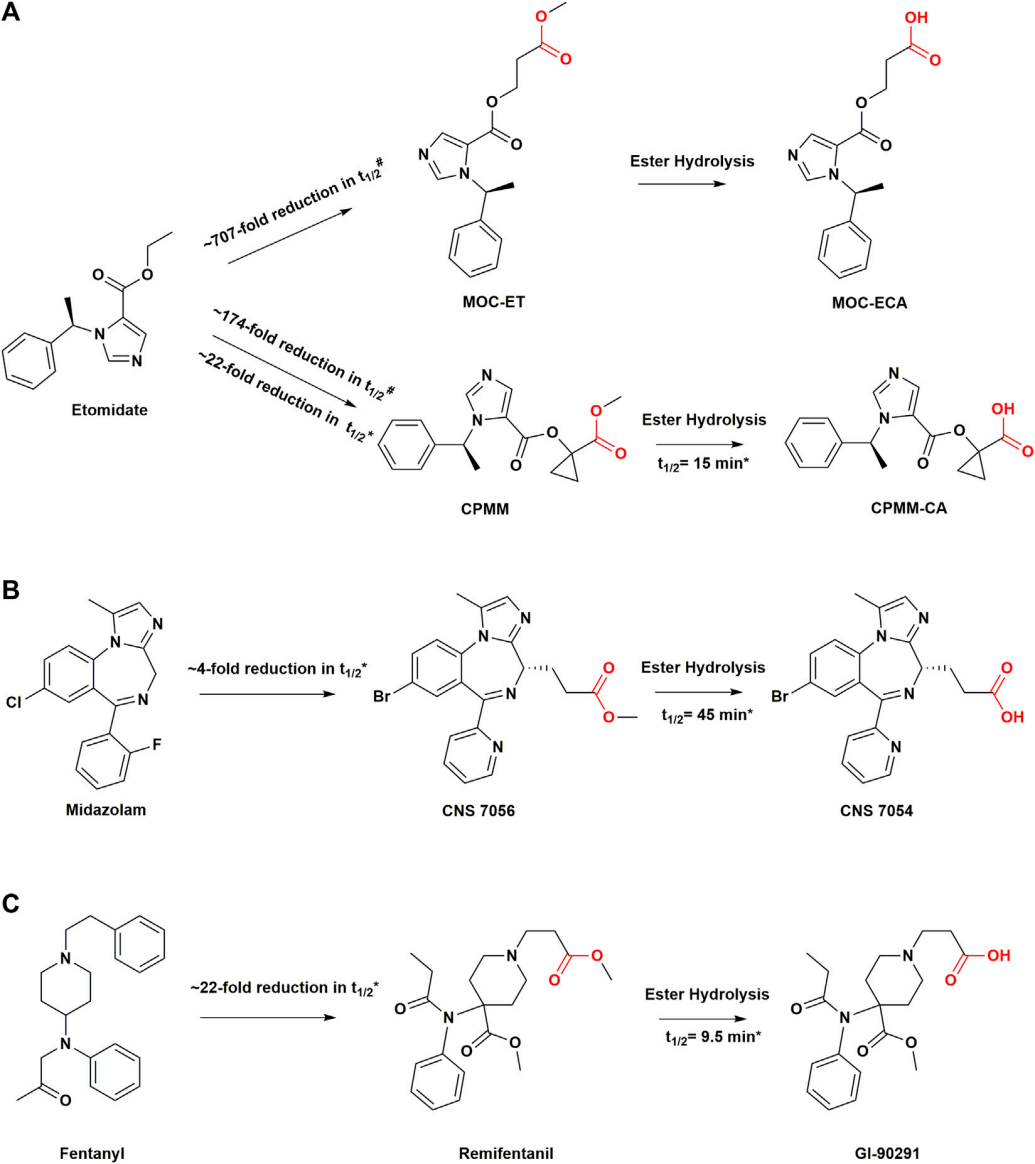
Structural modifications in sedatives and analgesics with soft drug design. **(A)** Soft analogs of etomidate and their metabolites. **(B)** Soft analog of midazolam and its metabolite. **(C)** soft analog of fentanyl and its metabolite. #Values are from measurements of *in vitro* metabolic half-lives in rat blood. *Values are from measurements of *in vivo* metabolic half-lives in human body. t_1/2_, half-life; MOC-ET, methoxycarbonyl etomidate; CPMM, cyclopropyl-methoxycarbonyl metomidate.

**FIGURE 4:**
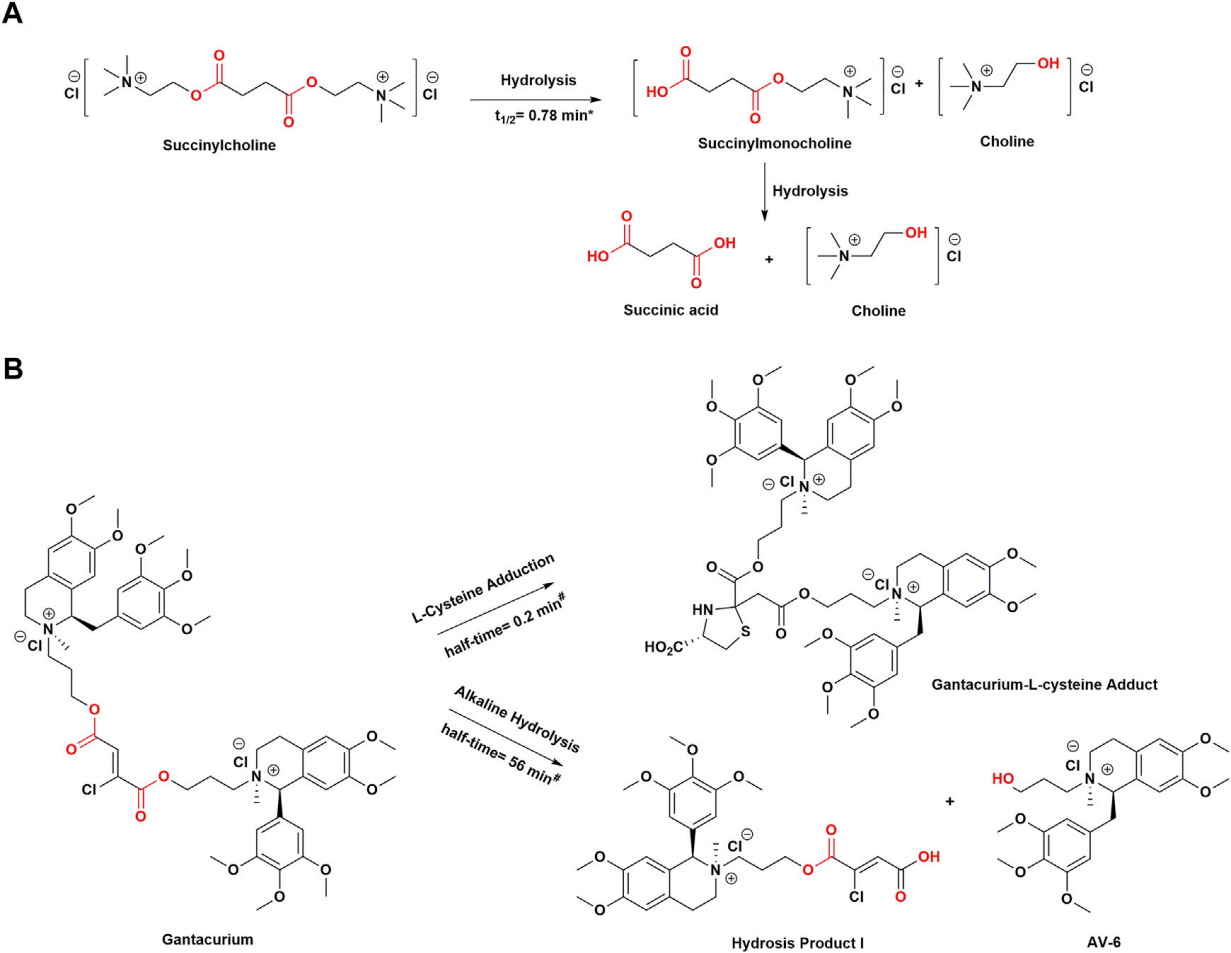
Structural modifications in muscle relaxants drugs with soft drug design. **(A)** Chemical structure of succinylcholine and its hydrolysis process. **(B)** Chemical structure of gantacurium and its breakdown products. *Values are from measurements of *in vivo* metabolic half-lives in human body. #Values are from measurements of *in vitro* reaction half-times in phosphate buffer at pH 7.4 and 37°C. t_1/2_, half-life.

Therefore, it is desirable that soft drugs are metabolized by a broad class of hydrolytic enzymes rather than undergoing oxidative metabolism. Mammalian carboxylesterases (EC 3.1.1.1) play an important role as enzymes for drug biotransformation and constitute a polygenic family with low substrate specificity (Laizure et al., 2013). Together with other carboxylate hydrolases, such as butyrylcholinesterase (BChE, EC 3.1.1.8) and arylesterase (ArE, EC 3.1.1.1.2), they effectively catalyze the hydrolysis of various chemicals containing functional groups, such as carboxylic acid esters, amides, and thioesters, to their respective free acids (Laizure et al., 2013; Di, 2019). As esterases are ubiquitous in mammals and widely expressed in various tissues, they provide a more reliable source of inactivation relative to metabolic enzymes that are expressed primarily in organs such as the liver and kidney, especially in critically ill patients with severely impaired liver and kidney function (Laizure et al., 2013). Therefore, many soft drug strategies are focused on hydrolysis by esterases. However, this does not imply that molecular structures containing ester bonds have the PK characteristics of rapid metabolism because steric hindrance around ester bonds can have a great impact on enzymatic reactions where the larger the steric hindrance, the longer the half-life (Buchwald and Bodor, 1999). Moreover, excessively rapid metabolism should be avoided to ensure the prolonged activity of the parent compound at the desired site (Bodor and Buchwald, 2000). Pharmaceutical development in anesthesiology has gravitated toward soft drugs because they cater to the high degree of control required over the rapidly changing clinical process allowing for a state of anesthesia that can be turned on immediately when desired and can be turned off in a controlled manner (Egan, 2009). It is desirable that not only should anesthetic drugs provide the advantage of predictable control, but also that the rapid metabolism of soft drugs should be independent of liver and kidney function and thus should not be altered by continuous infusion and multiple intermittent repeated doses. The drug development in anesthesia has therefore gradually focused on designing soft drugs for sedation, analgesia, and muscle relaxation in recent years (Figure 3, Figure 4) (Egan, 2009; Birgenheier et al., 2020). Since esmolol was first marketed as an ultra short-acting *ß*-blocker in the early 1980s (Erhardt et al., 1982), this aforementioned successful drug design approach has been rapidly applied to opioid analgesics, benzodiazepine scaffolds, and other ester derivatives (Feldman et al., 1991; James et al., 1991; Stafford et al., 2002). In most cases, such drugs are rapidly metabolized, and the pharmacological activity of the corresponding metabolites is one or more orders of magnitude lower than that of the parent drug (Shaffer et al., 1988; Westmoreland et al., 1993; Kilpatrick et al., 2007). Some well-designed soft drugs have been approved for clinical use; however, many novel soft drug development programs have discontinued such as those of novel etomidate and propanidid soft drug analogs (Egan et al., 2012; Husain et al., 2012). Therefore, ongoing attempts to develop soft drugs in the field of anesthesia will need to see some type of success in order to provide options for subsequent drug development.

## Sedatives

### Etomidate and Analogues

Etomidate is an ester-containing, short-acting imidazole-type derivative discovered in 1964 (Godefroi et al., 1965). As a nonbarbiturate intravenous general anesthetic, it has outstanding pharmacological characteristics and causes rapid induction of the state of anesthesia. Etomidate is hemodynamically stable during anesthesia and has little effect on respiratory effects (Morgan et al., 1975). Etomidate is inactivated by hepatic esterases, which leads to the formation of corresponding carboxylic acid (ET-acid) (Van Hamme et al., 1978). Although etomidate also contains a carboxylic ester structure that can be hydrolyzed to carboxylic acids by hepatic esterases, it is a poor substrate for these esterases (Van Hamme et al., 1978). Owing to the structural proximity of the ester bond and the imidazole ring, the steric hindrance of hydrolysis increases, prolonging the duration of action with a terminal metabolic half-life of approximately 2–5 h (Van Hamme et al., 1978; Forman, 2011). In critically ill patients, induction with etomidate could increase mortality by inhibiting adrenocortical steroid synthesis (Ledingham and Watt, 1983; Wagner and White, 1984). Studies have shown that etomidate could cause adrenocortical function of up to 6-8 h from a single administration, and more than 24 h from continuous infusion (Allolio et al., 1984; Wanscher et al., 1985), severely limiting its clinical application. A valid explanation for this could be its high affinity for 11β-hydroxylase and the cholesterol side-chain cleavage enzyme, which are the key enzymes involved in steroidogenesis (de Jong et al., 1984; Pejo et al., 2016). Therefore, it has been hypothesized that “soft” etomidate derivatives would ameliorate this side effect since the drugs would be rapidly metabolized (McGrath and Raines, 2018).

Methoxycarbonyl etomidate (MOC-etomidate) is the first soft analog of etomidate, which can be rapidly hydrolyzed by nonspecific esterase activity because of its metabolically labile ester (Cotten et al., 2009). The main metabolite is methoxycarbonyl etomidate carboxylic acid (MOC-ECA). Owing to the two-carbon spacer, which increases the length between the labile ester moiety and the imidazole ring in MOC-etomidate, the steric hindrance of ester hydrolysis decreases significantly (Cotten et al., 2009). Therefore, compared with etomidate, MOC-etomidate can be deactivated by rapid hydrolysis *in vivo*, with a half-life of only a few minutes (Husain et al., 2012). As a result, it maintains the hypnotic effect with a shorter duration of action and is devoid of the suppression of adrenocortical function after a single bolus (Cotten et al., 2009). It is also demonstrated that PK modifications can reduce the inhibitory effect of etomidate analogs on adrenocortical function (Cotten et al., 2009). However, studies have shown that MOC-ECA accumulates gradually over time during continuous infusion, leading to the suppression of electroencephalographic activity (Ge et al., 2012). The related hypothesis could be that ultrafast metabolism results in marked context sensitivity with a slow clearance of metabolites in the brain in which case even minimal active metabolites may accumulate to concentrations sufficient to produce significant pharmacological effects despite the metabolite potency of MOC-ECA being 350-fold lower than that of MOC-etomidate (Ge et al., 2012; Pejo et al., 2012).

Cyclopropyl-methoxycarbonyl-metomidate (CPMM, currently known as ABP-700) is a second-generation soft analog of etomidate, a member of the MOC-etomidate analogs family, with an optimal onset and offset profile (Husain et al., 2012). The researchers concluded that incorporating different aliphatic groups on the two-carbon intervals of MOC-etomidate and the introduction of steric hindrance could help reduce the rate of ester bond hydrolysis and prolong the duration of anesthesia. This strategy was based on previous studies which found that the insertion of large chemical groups near metabolically unstable ester bonds slowed down the rate of ester bond hydrolysis (Buchwald, 2001; Buchwald and Bodor, 2002). Therefore, although ABP-700 contains an ester bond which can be metabolized to a carboxylic acid metabolite by nonspecific esterases, it is metabolized as a soft drug more slowly than MOC-etomidate (Pejo et al., 2016). Compared to MOC-etomidate, ABP-700 has an additional aliphatic group, namely, cyclopropyl, between the etomidate backbone and the labile ester, which slows down the metabolic rate (Husain et al., 2012). At the same time, such a structure leads to unexpected potency, which is approximately an order of magnitude higher than that of MOC-etomidate (Husain et al., 2012). These chemical modifications combine optimized PK and PD properties while improving the overall therapeutic index.

Preclinical studies in rodent models have shown that the ABP-700 group recovered faster than the etomidate group and that the recovery time was independent of the infusion time. Furthermore, the electroencephalographic burst suppression ratio (BSR) was reversed within minutes after discontinuation of the closed-loop infusion that lasted 2 h (Pejo et al., 2012). Cortisol concentrations in beagles did not differ from those of beagles in the propofol group after 2 h of continuous infusion of ABP-700 in response to extraneous corticotropic hormone stimulation (Campagna et al., 2014). Clinical studies on the safety and efficacy of ABP-700 have shown that ABP-700 retains the desirable properties of etomidate with hemodynamic stability and no respiratory depression (Struys et al., 2017; Valk et al., 2018). However, patients in the ABP-700 group often experienced excitatory phenomena and abnormal involuntary muscle movement excitation at clinical doses (Valk et al., 2018), which led to discontinued development by The Medicines Company in 2017. Recent studies have shown no correlation between involuntary muscle movement and epilepsy (Valk et al., 2019), although, disinhibitory effects of the Bispectral Index (BIS) are associated with involuntary muscle movements (Valk et al., 2021). The mechanisms underlying clinical excitation are still unclear, and ABP-700 was restarted in 2020 with funding by Mass General Brigham (Boston, MA, United States), which still needs further investigation (Valk and Struys, 2021).

### Benzodiazepines

Benzodiazepines constitute a class of psychotropic drugs with hypnotic, sedative, anti-anxiety, and anterograde amnesia properties that have been widely used in clinical practice. Midazolam remains one of the most commonly used sedatives. However, its elimination half-life is approximately 1.5–3 h and it has a prolonged sedation time due to its production of active metabolites and dependence on liver metabolism (Allonen et al., 1981; Smith et al., 1981). Moreover, the cytochrome P450 enzyme has individual differences, making the sedative duration of midazolam unpredictable (Kanto, 1985; Wandel et al., 1994). Remimazolam, formerly known as CNS 7056, is an ester-modified benzodiazepine derivative with a rapid offset of drug effect. It has a typical pharmacological profile of benzodiazepines while exhibiting the soft PK properties of remifentanil (Goudra and Singh, 2014). Pharmacodynamically, remimazolam is similar to midazolam and acts on gamma-aminobutyric acid (GABA_A_) receptors which can be antagonized by the benzodiazepine antagonist flumazenil (Kilpatrick et al., 2007). Similar to remifentanil, its metabolism is organ-independent, and it can be administered to patients with renal or hepatic impairment (Stöhr et al., 2021). The carboxylic acid ester bond in the molecular structure of remimazolam is readily inactivated *in vivo* by nonspecific esterases in the blood and tissues. Its carboxylic acid metabolite, CNS 7054, has 1/320 to 1/410 times the affinity for the benzodiazepine receptor than that of remimazolam (Kilpatrick et al., 2007). These characteristics lend remimazolam a shorter action time (<10 min) than midazolam, with an elimination half-life of only 0.75 h (Antonik et al., 2012). Clinical trials have shown that remimazolam has a rapid onset of action and rapid recovery after sedation. In phase II and III clinical trials, remimazolam was safely used in outpatient gastrocolonoscopy, and patients recovered more quickly than those treated with midazolam (Borkett et al., 2015; Pambianco et al., 2016; Rex et al., 2018). Remimazolam demonstrates deeper sedation and quicker recovery than midazolam during continuous infusion in healthy Chinese participants (Sheng et al., 2020). It demonstrated a controllable pharmacological effect profile even after long-lasting continuous infusion, and a high clearance (Lohmer et al., 2020; Schüttler et al., 2020). The sedative effect of this drug is comparable to that of propofol, with rapid peak sedation within 1–2 min and only moderate hemodynamic side effects (Goudra and Singh, 2014; Birgenheier et al., 2020). Currently, remimazolam is seen as the future of sedatives, which has already received regulatory approval in Japan for general anesthesia in adults. It has been approved by the United States Food and Drug Administration (US FDA) for the induction and maintenance of procedural sedation (Keam, 2020). A more complete picture of the clinical potential of remimazolam will be available in the coming years as the drug moves from late-stage development to more widespread postmarketing use.

## Analgesics

As an ultra short-acting MOR agonist, remifentanil synthesized by Feldman et al., has a strong analgesic effect (Feldman et al., 1991; Bürkle et al., 1996). The introduction of a methyl ester group on the N-acyl side chain of the piperidine ring improves sensitivity to esterase hydrolytic metabolism (Feldman et al., 1991). Studies have shown that the metabolic clearance rate of remifentanil is faster than that of liver blood flow, indicating that the metabolism of remifentanil is independent of the liver (Glass et al., 1999). It can be easily metabolized by nonspecific plasma and tissue esterases into the carboxylic acid metabolite GI-90291 (Glass et al., 1999). GI-90291 has a potency of 1/300 to 1/1000 times that of remifentanil and is inactive at clinical doses. The renal excretion rate of this metabolite has been estimated to be over 80% (Glass et al., 1993). Remifentanil has a short onset of action of 1–2 min and a terminal half-life of 10–20 min (Westmoreland et al., 1993). After continuous infusion for 3 h, the time-dose-related half-life of remifentanil was only approximately 3 min, preventing undesirable accumulation (Kapila et al., 1995). This also explains why hydrolases are popular for soft drug inactivation. In the design of etomidate analogs, the idea of inserting two CH_2_ groups between the labile ester group and the imidazole ring was also derived from the structure of remifentanil (Cotten et al., 2009). Remifentanil had the same potency as fentanyl in a rat model of tail removal response, and the duration of action was four times shorter (Feldman et al., 1991). The potency of remifentanil was 20–30 times higher than that of alfentanil in both healthy adult volunteers and those who underwent surgery (Egan et al., 1996; Scott and Perry, 2005). Remifentanil, as an effective opioid analgesic, has been widely used for perioperative pain management (Wilhelm and Kreuer, 2008).

## Muscle Relaxants

Succinylcholine, an accidental soft drug introduced in the 1950s, is the only currently available depolarizing muscle relaxant (DMR) with favorable PK properties (Thesleff et al., 1952). It contains two acetylcholine molecules linked together by methyl acetate (Jonas and Hunter, 2004). Succinylcholine is normally rapidly degraded in plasma by pseudocholinesterase (PChE) to succinylmonocholine, succinic acid, and choline (Viby-Mogensen, 1980; Curran et al., 1987). However, given that PChE is synthesized in the liver and is present in the plasma, liver-related diseases may decrease enzyme activity and prolong the duration of associated neuromuscular block. In addition, patients with PChE deficiency are incapable of metabolizing suxamethonium, resulting in prolonged apnea (Al-Emam, 2021). In general, the onset time of succinylcholine is within 60 s and lasts for 4–6 min when administered to patients with normal plasma PChE activity (Alvarellos et al., 2015). Thus, because of its short half-life, it is often used in clinical procedures requiring short periods of muscle relaxation, such as endotracheal intubation, fiberoptic bronchoscopy, and electroconvulsive therapy (Sluga et al., 2005; Li et al., 2016). However, it suffers some serious side effects, such arrhythmia, hyperkalemia, increased intraocular or gastric pressure, and sometimes even malignant hyperthermia, precluding it from being the “ideal” ultra short-acting DMR (Galindo and Davis, 1962; Walton and Farman, 1973; Meyers et al., 1978).

In the past few decades, there have been some nondepolarizing muscle relaxants (NDMRs) that may be used instead of succinylcholine, such as short-acting gantacurium. Gantacurium chloride, formerly recognized as GW280430A, is a rapid onset NDMR (Belmont et al., 2004; de Boer and Carlos, 2018). As a bis-tetrahydroisoquinolinium chlorofumarate, it is a single isomer such as cisatracurium, whereas atracurium and mivacurium consist of a mixture of isomers (Boros et al., 1999). In preclinical and clinical trials, gantacurium was regarded as a promising candidate because it seemed to have a nearly identical kinetic profile to succinylcholine. In human volunteers, its effect onset time was less than 3 min, which was capable of being shortened to approximately 1.5 min by increasing the dose to four times the effective dose of 95% (ED_95_) with a duration of action of 15 min (Belmont et al., 2004). There are two routes of inactivation that are unrelated to PChE activity. One is a slow process, in which it is metabolized by alkaline ester hydrolysis in plasma, and the other is a fast process that involves adduction of the amino acid cysteine (L-cysteine) to saturate the fumarate double bond (Savarese et al., 2004). The latter method of chemical degradation most likely accounts for its ultrashort duration of effect. Moreover, the unique means of elimination involve neither the kidney nor the liver, and the metabolites of gantacurium are pharmacologically inactive (Belmont et al., 2004). Numerous studies have indicated that L-cysteine adduction can reverse neuromuscular blockade of gantacurium and its analogs (CW 002 and CW 011), which is the same as sugammadex reversal of rocuronium (Savarese et al., 2010; Sunaga et al., 2010).

## Conclusion

From pharmacological and toxicological perspectives, metabolic considerations in the drug design process help improve PK/PD and safety profiles. For both prodrugs and soft drugs, the applied strategies are driven by unmet medical needs and are used to overcome undesirable drug properties to achieve optimal clinical use. In fact, from a broad perspective, prodrugs and soft drugs seem to be two extremes of a continuum of possibilities (Stańczak and Ferra, 2006). Prodrugs design is a very useful approach for improving the drug-like properties of a molecule to circumvent formulation and delivery difficulties. In the case of the soft drug approach, the retrometabolic drug design strategy allows a predictable metabolic route via a single inactivation. Prodrug design strategies have a wide range of applications, and soft drug design represents an approach that meets the unique needs of modern anesthesia practice.

In recent years, new drug development programs for the analogs of anesthetics have resulted in only a handful of compounds with market approval (Liu et al., 2016; Keam, 2020). For soft sedative-hypnotics, abnormal excitatory activity has been the main reason for discontinuing the development programs. This is the case for the etomidate and propanidid soft drug analogs. To compete with existing drugs, novel anesthetic drugs should possess a high therapeutic index and minimal side effects to optimize the benefit/risk ratios in patients.

Researchers have gradually applied artificial intelligence-assisted drug design strategies to drug metabolism studies in recent years (Wang et al., 2019; Smith, 2022). As enzymes (usually cytochrome P450) are essential for drug metabolism, the three-dimensional crystal structures of various enzymes and carrier proteins have been analyzed, the results of which have provided the basis for structural information (Pochapsky and Pochapsky, 2019). This would render the prediction of interactions achievable at the beginning of drug design (Smith, 2022). Although there are several challenges and failures in drug development, these experiences have driven the development of new compounds. In this review, the important roles of drug metabolism and pharmacokinetics strategies in drug design are emphasized and expounded through examples of various prodrugs and soft drugs in anesthesia. The rational use of these strategies will help develop more effective and safer drugs in the future.
